# Clinicopathological Characteristics and Prognostic Prediction in Pseudomyxoma Peritonei Originating From Mucinous Ovarian Cancer

**DOI:** 10.3389/fonc.2021.641053

**Published:** 2021-04-21

**Authors:** Fengxian Fu, Xulan Ma, Yiyan Lu, Hongbin Xu, Ruiqing Ma

**Affiliations:** ^1^Department of Gynecology, Aerospace Center Hospital, Beijing, China; ^2^Department of Pathology, Aerospace Center Hospital, Beijing, China; ^3^Department of Myxoma, Aerospace Center Hospital, Beijing, China

**Keywords:** pseudomyxoma peritonei, mucinous ovarian cancer, prognostic prediction, overall survival (OS), cytoreductive surgery (CRS) and hyperthermic intraperitoneal chemotherapy (HIPEC)

## Abstract

**Objective:**

To describe the clinicopathological characteristics of mucinous ovarian cancer (MOC)-derived pseudomyxoma peritonei (PMP) and identify prognostic factors for survival.

**Methods:**

Medical records from patients with MOC-derived PMP who attended the Aerospace Center Hospital, Beijing, China between January 2009, and December 2019 were retrospectively reviewed. Survival analysis was performed with the Kaplan-Meier method, the log-rank test, and a Cox proportional hazards model.

**Results:**

Cytoreductive surgery (CRS) and hyperthermic intraperitoneal chemotherapy (HIPEC) for PMP originating from MOC were performed on 22 patients, who had a median age of 52 years at the time of surgery. At the last follow-up in June 2020, 9 (41%) patients were still alive. Median OS was 12 months (range, 1 to 102 months), and the 2-, 3-, and 5-year survival rates were 23, 9, and 5%, respectively.

**Conclusion:**

Histopathologic subtype and PCI may be applied as predictors of prognosis in patients with MOC-derived PMP. Patients with high-grade disease could benefit from completeness of cytoreduction (CCR) 0/1.

## Introduction

Ovarian cancer is the second most common gynecological malignancy following uterine corpus cancer, and the most pernicious neoplasm in the female reproductive system (1). Epithelial ovarian cancer (EOC) is the most common ovarian malignancy, of which primary mucinous ovarian cancer (MOC) is a rare histotype, accounting for 3 to 5% of EOC cases (2). Early-stage MOC is associated with a good prognosis; however, advanced-stage MOC is relatively resistant to first-line chemotherapy, and patients have worse survival compared to other EOC histotype (3).

MOC may metastasize to the peritoneum giving rise to pseudomyxoma peritonei (PMP), a rare clinical entity characterized by diffuse intra-abdominal gelatinous ascites with mucinous implants on peritoneal surfaces and no obvious invasion of underlying tissues. The estimated incidence of PMP is 1–2 per million per year (4). PMP usually originates from a primary mucinous tumor of the appendix, but other primary localizations have been reported.

MOC-derived PMP is an uncommon disease entity, which lacks clinical data and information on patient prognosis. The objective of this retrospective analysis of 22 patients with MOC-derived PMP was to describe the clinicopathological characteristics of MOC-derived PMP and identify prognostic factors for survival. Findings from this study will inform the management of patients with MOC-derived PMP.

## Materials and Methods

### Ethical Approval

The Ethics Committee of the Aerospace Center Hospital, Beijing, China (No. 20161109-ST-07) approved this study. Written inform consent for the use of clinical data and images was obtained from all included patients.

### Patient Population

Medical records from a database of patients with PMP who attended the Aerospace Center Hospital, Beijing, China between January 2009 and December 2019 were retrospectively reviewed. Inclusion criteria were: (1) diagnosis of MOC-derived PMP on histology and histopathologic subtype confirmed by two experienced pathologists; and (2) treatment with cytoreductive surgery (CRS) and hyperthermic intraperitoneal chemotherapy (HIPEC). Exclusion criteria were: (1) PMP derived from other organs or disease (e.g., colon, urachus, and pancreas); (2) loss to follow-up; (3) incomplete medical records; or (4) history of other severe organic disease. A total of 22 patients were included in the final analysis.

#### Surgery Treatment

**Figure 1:**
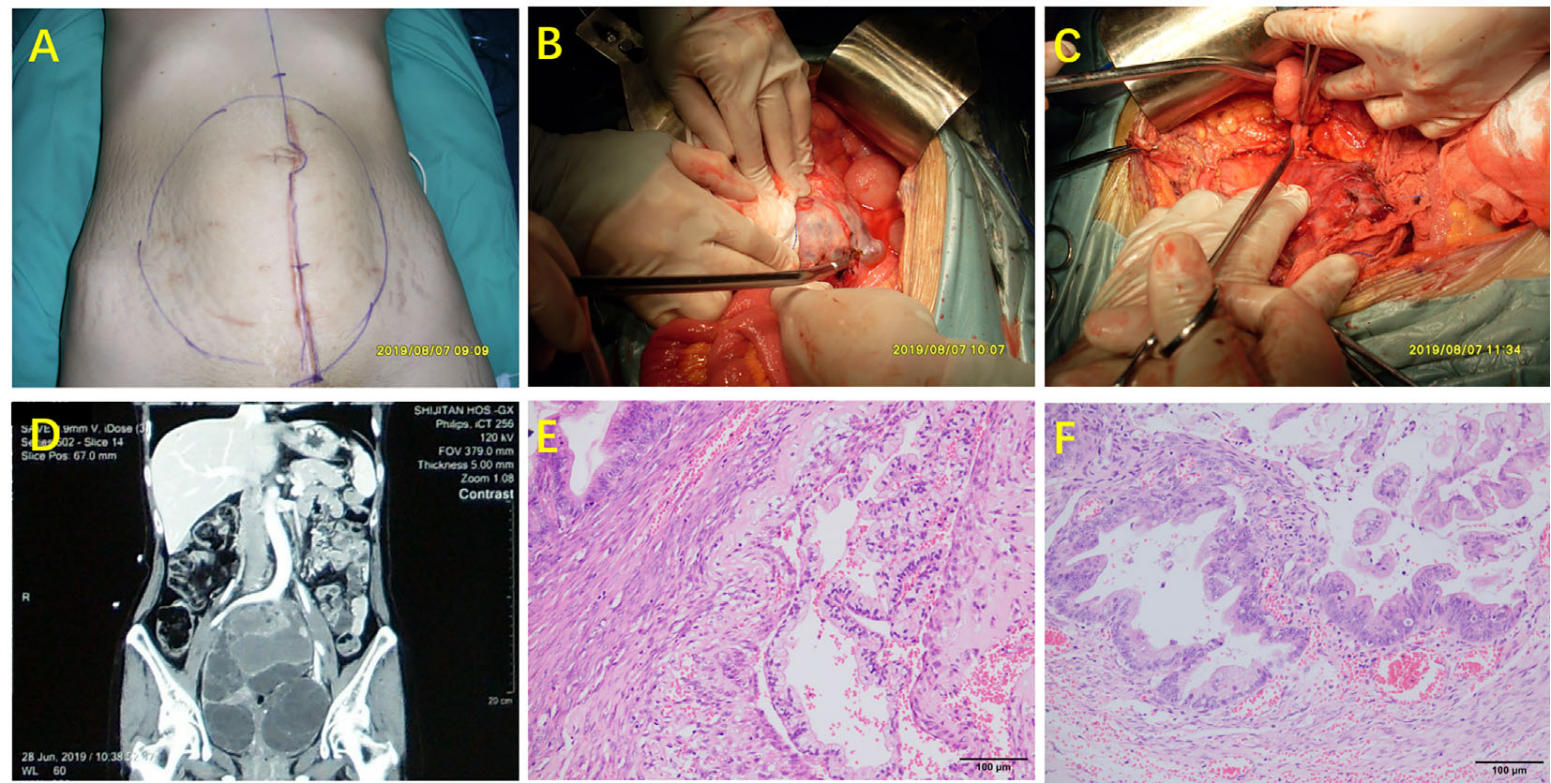
Surgical procedure (complete cytoreductive surgery lasted about 10 h) and pathology of MOC-derived PMP. **(A)** Status of the abdomen preoperatively; **(B)** Identification of ovarian involvement; **(C)** The appendix was not involved; **(D)** CT scan prior to CRS; **(E)** H&E staining of mucinous ovarian tumor (200× magnification; **(F)** H&E staining of appendix (200× magnification).

Patients underwent CRS to remove visible tumors if any (5) (**Figure 1**). HIPEC was conducted in all included patients for 60 min using a closed-abdomen technique with cisplatin 60~80 mg and an extracorporeal device that maintained intraabdominal temperature between 40 and 42°C.

#### Study Parameters

The analysis included the following clinicopathological parameters: gender, age at diagnosis of MOC-derived PMP, time from diagnosis of MOC to CRS for PMP, status, overall survival (OS), survival rate (2/3/5-year), history of (with/without) previous systemic chemotherapy (PSC), prior surgical score (PSS), intraoperative peritoneal cancer index (PCI), completeness of cytoreduction (CCR), preoperative serum CEA/CA125/CA19-9 elevation, intraoperative HIPEC, pathological grade, history of (presence/absence) lymph node metastasis, history of (with/without) prior appendectomy, and follow-up time.

Among these characteristics, PSS was scored on a scale from 0 to 3, where PSS-0 was no surgery or biopsy had been performed for cancer, PSS-1 was surgery had been performed in one abdominal region, PSS-2 was surgery had been performed in two to five regions, and PSS-3 was surgery had been performed in >5 regions (6). The PCI was determined intraoperatively where tumor volume and extent of peritoneal dissemination in each of 13 abdominopelvic regions (nine anatomical regions in the abdomen and four segments in the small bowel) were scored on a scale from 0 to 3 and summed (5). Residual disease following CRS was scored according to the CCR on a scale from 0 to 3, where CCR-0 was no macroscopic residual cancer remained, CCR-1 was residual tumor nodules < 2.5 mm remained, CCR-2 was nodules between 2.5 mm and 2.5 cm remained, and CCR-3 was persistent tumor nodules > 2.5 cm remained (7). Pathological diagnosis was classified into four categories, according to the 2016 Peritoneal Surface Oncology Group International (PSOGI) criteria (8), as acellular mucin (AC), low-grade mucinous carcinoma peritonei (LG-MCP), high-grade mucinous carcinoma peritonei (HG-MCP), or high-grade mucinous carcinoma peritonei with signet ring cells (HGMC-S). OS was calculated from the date of CRS and HIPEC to the time of death or the last follow-up.

The immunohistochemistry information of CK7, CK20, CDX-2, MUC-2, and PAX-8 were recorded. We classify the pathological type as the intestinal borderline mucinous tumor (IBMT) when CDX-2 is positive, as the positive rate of CDX-2 in intestinal tumors is 100% (9, 10). When both CDX-2 and PAX-8 are positive, we need to combine the expression of MUC-2. If MUC-2 was negative, we preferred the endocervical mucinous unilateral tumor. When MUC-2 was positive, it tends to be IBMT. PAX-8 has high specificity but low sensitivity in differentiating ovarian mucinous tumors from appendiceal mucinous tumors. In the present study, if PAX-8 was clearly positive, we tended to classify it as endocervical mucinous unilateral tumor. If PAX-8 was negative, and CDX-2 was negative, then we need to combine the MUC-2 expression pattern. If MUC-2 was negative, we tend to classify it as the endocervical mucinous unilateral tumor. If MUC-2 was positive, the patient tends to be IBMT.

### Statistical Analysis

Statistical analyses were conducted using SPSS 24.0 (IBM Corporation, Armonk, NY, USA). Continuous data are presented as medians and range. Categorical data are presented as number and percentages. Univariate survival analysis was performed with the Kaplan-Meier method and the log-rank test. Statistically significant variables were included in a multivariate analysis, which used a Cox proportional hazards model to identify independent prognostic factors for survival. All live patients were censored. P < 0.05 was considered statistically significant.

## Results

### Clinicopathological Characteristics

From January 2009 to December 2019, 963 patients with PMP were included in the database established by the Aerospace Center Hospital, Beijing, China. Of these, 888 (92%) patients had appendix derived PMP. A total of 584 patients with PMP had ovarian involvement, including 549 patients with secondary involvement of the ovary, and 35 patients with MOC-derived PMP. Among the patients with MOC-derived PMP, 13 of them excluded due to incomplete medical records and/or loss of the follow-up. Finally, 22 patients were included in the final analysis.

Demographic and clinical characteristics of the 22 included patients are presented in **Table 1**. Patient’s median age at MOC diagnosis was 47 years (range, 29 to 67 years). Patient’s median age at CRS and HIPEC for PMP originating from MOC was 52 years (range, 33 to 67 years). Median time from diagnosis of MOC to CRS and HIPEC for PMP was 28 months (range, 0 to 120 months). Median follow-up after CRS and HIPEC was 31 months (range, 6 to 108 months). At the last follow-up in June 2020, 9 (41%) patients were still alive, median OS was 12 months (range, 1 to 102 months), and the 2-, 3-, and 5-year survival rates were 23, 9, and 5%, respectively. PSC was performed in 10 (45%) patients before CRS and HIPEC, and PSS was 0/1, 2, and 3 in 6 (27%), 12 (55%), and 4 (18%) patients, respectively. Serum CA125, CA19-9, and CEA were elevated in 11 (50%), 14 (64%), and 5 (23%) patients, respectively.

**Table 1:** Patients’ clinical and demographic data (n = 22).

Characteristics	No. of Patients
Gender	
Female	22 (100%)
Male	0
MOC-age at diagnosis (years)	
Median (range)	47 (29–67)
<50	13 (59%)
≥50	9 (41%)
PMP-age at diagnosis (years)	
Median (range)	52 (33–67)
<50	10 (45%)
≥50	12 (55%)
Time from MOC diagnosis to CRS and HIPEC (months)	
Median (range)	28 (0–120)
PSC	
Yes	10 (45%)
No	12 (55%)
PSS	
0/1	6 (27%)
2	12 (55%)
3	4 (18%)
Elevated serum tumor markers	
CA125	11 (50%)
CA19-9	14 (64%)
CEA	5 (23%)
PCI	
Median (range)	20 (5–39)
<20	11 (50%)
≥20	11 (50%)
CCR	
Median (range)	2 (0–3)
0/1	11 (50%)
02-Mar	11 (50%)
HIPEC	
Yes	22 (100%)
No	0
Histopathologic subtype	
LGMC	9 (41%)
HGMC	13 (59%)
Immunohistochemistry results	
**CK7**	
Positive	18 (82%)
Partial positive	3 (14%)
Negative	1 (4%)
**CK20**	
Positive	10 (45%)
Partial positive	6 (27%)
Negative	6 (27%)
**CDX2**	
Positive	10 (45%)
Partial positive	1 (4%)
Negative	11 (50%)
**MUC-2**	
Positive	7 (32%)
Weakly positive	2 (9%)
Negative	12 (55%)
Missing	1 (4%)
**PAX-8**	
Positive	6 (27%)
Weakly positive	4 (18%)
Negative	1 (4%)
Missing	1 (4%)
Primary ovarian tumor	
IBMT	10 (45%)
Endocervical mucinous unilateral tumor	12 (55%)

MOC, mucinous ovarian carcinoma; PMP, pseudomyxoma peritonei; CRS, cytoreductive surgery; PSC, previous systemic chemotherapy; PSC, prior surgical score; PCI, peritoneal cancer index; CCR, completeness of cytoreduction; HIPEC, hyperthermic intraperitoneal chemotherapy; LGMC, low-grade mucinous carcinoma peritonei; HGMC, high-grade mucinous carcinoma peritonei; IBMT, intestinal borderline mucinous tumors. In bold: CK7, Cytokeratin 7; CK20, Cytokeratin 20; CDX2, Caudal Type Homeobox 2; MUC-2, Mucin-2; PAX-8, Paired Box 8.

Intraoperative PCI was <20 and ≥20 in 11 (50%) and 11 (50%) patients, respectively. CCR score was 0/1 and 2/3 in 11 (50%) and 11 (50%) patients, respectively. Pathological diagnosis showed 13 (59%) patients had HGMC and 9 (41%) patients had LGMC. Two (9%) patients had lymph node metastasis, and no other space-occupying lesions of pelvic and abdominal organs were observed in all cases. For all the included patients, 19 were pathologically confirmed as chronic appendicitis (6 appendixes were removed in our hospital, and 13 cases were removed in other hospitals). Surgical exploration showed a totally normal appendix appearance in two cases. In addition, one appendix could not be identified due to intraperitoneal surgery adhesion. However, the primary source was approved as an endocervical mucinous unilateral tumor by the immunohistochemistry examination. There were 10 (45%) cases of the IBMT and 12 (55%) cases of the endocervical mucinous unilateral tumor based on the immunohistochemistry results. In the cases of IBMT, we found no evidence of teratoma after extensive sampling in all included patients (mean 42 blocks ranging from 21 blocks to 68 blocks).

### Univariate Analysis

**Figure 2:**
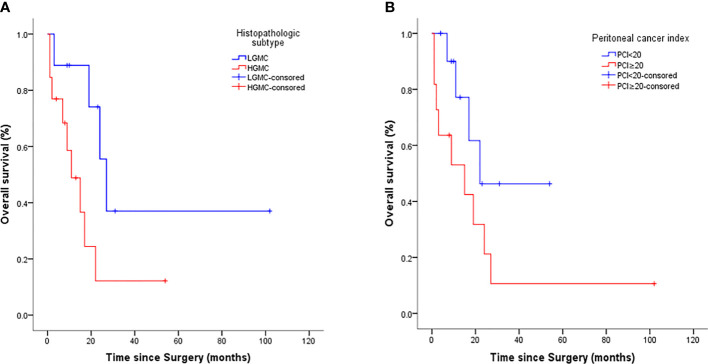
Kaplan-Meier survival curves for histopathologic subtype **(A)** and peritoneal cancer index **(B)** (n = 22).

Prognostic factors for OS on univariate analysis are presented in **Table 2**. Among all included patients, the histopathologic subtype of MOC-derived PMP (HGMC *vs.* LGMC) was prognostic for OS by the log rank test (P = 0.039). Among patients stratified by histopathologic subtype of MOC-derived PMP (HGMC or LGMC), PCI (<20 *vs.* ≥20, P = 0.026), and CCR (0/1 *vs.* 2/3, P = 0.031) were prognostic for OS by the log rank test in patients with HGMC (**Figure 2**).

**Table 2:** Univariate analysis of OS after CRS and HIPEC (n = 22).

Variables	Log-Rank P value of Univariate analysis					
	Overall		LGMC		HGMC	
	HR (95%CI)	P value	HR (95%CI)	P value	HR (95%CI)	P value
MOC-Age (<50 *vs.* ≥50, years)	1.496 (0.504–4.439)	0.459	4.143 (0.348–49.28)	0.074	0.375 (0.062–2.249)	0.118
PMP-Age (<50 *vs.* ≥50, years)	1.338 (0.439–4.078)	0.593	2.333 (0.121–45.17)	0.405	0.625 (0.156–2.500)	0.434
PSC (Yes *vs.* No)	0.698 (0.234–2.083)	0.51	0.298 (0.042–2.124)	0.259	0.841 (0.222–3.189)	0.792
PSS (0/1 *vs.* 2/3)	1.201 (0.353–4.088)	0.757	2.193 (0.121–39.69)	0.469	0.699 (0.186–2.632)	0.598
CA125 (<35 *vs.* ≥35, U/ml)	0.558 (0.188–1.659)	0.295	0.532 (0.068–4.167)	0.512	1.004 (0.251–4.019)	0.995
CA19-9 (<37 *vs.* ≥37, U/ml)	0.586 (0.186–1.849)	0.404	0.000 (−1.000–1.000)	0.176	0.833 (0.216–3.213)	0.782
CEA (<10 *vs.* ≥10, ng/ml)	1.289 (0.416–3.988)	0.661	0.572 (0.075–4.367)	0.614	0.244 (0.005–11.51)	0.125
PCI (<20 *vs.* ≥20)	0.390 (0.131–1.158)	0.1	0.000 (−1.000–1.000)	0.147	0.156 (0.030–0.798)	0.026*
CCR (0/1 *vs.* 2/3)	0.683 (0.229–2.039)	0.483	0.000 (−1.000–1.000)	0.176	0.080 (0.008–0.791)	0.031*
Histopathologic subtype (LGMC *vs.* HGMC)	0.323 (0.108–0.972)	0.039*				

MOC, mucinous ovarian carcinoma; PMP, pseudomyxoma peritonei; PSC, previous systemic chemotherapy; PSS, prior surgical score; PCI, peritoneal cancer index; CCR, completeness of cytoreduction; LGMC, low-grade mucinous carcinoma peritonei; HGMC, high-grade mucinous carcinoma peritonei. *P<0.05.

## Discussion

PMP is a rare malignant growth characterized by mucinous ascites in the peritoneal cavity. In 1842, Rokitansky first described mucocele of the appendix (11). In 1884, Werth applied the term PMP to a mucinous tumor of the ovary (12). PMP is usually secondary to an appendiceal mucinous neoplasm but may be associated with mucinous tumors of other primary sites, including the ovaries. PMP of non-appendiceal origin may have a worse prognosis than PMP originating from an appendiceal tumor as the underlying pathology is more likely to resemble a mucinous adenocarcinoma (4).

This study described the clinicopathological characteristics of MOC-derived PMP and identified independent predictors of OS. From January 2009 to December 2019, 963 patients with PMP attended the Aerospace Center Hospital, Beijing, China. PMP predominantly originated in the appendix, which is consistent with other studies (13). Thirty-five patients had MOC-derived PMP; of these, 22 patients were included in our analyses.

The incidence of primary MOC is rare, and a revision of diagnostic criteria for mucinous EOC has seen a reduction in the incidence of MOC from >10% of EOC cases to 3–5% of EOC cases (2). Approximately 80% of MOC are metastatic, originating from primary tumors of the gastrointestinal tract (45%), pancreas (20%), cervix and endometrium (18%), or breast (8%) (14). Metastatic mucinous carcinoma may be misdiagnosed as primary MOC. A comprehensive evaluation of patients to exclude metastatic tumors masquerading as MOC is an important step in the diagnosis of primary MOC.

In the present study of MOC-derived PMP, the histological origin of PMP was confirmed by two experienced pathologists. The median age of patients at the time of diagnosis of MOC was 47 years (range, 29 to 67 years). Patients were treated with CRS and HIPEC at a median age of 52 years (range, 33 to 67 years). Median time from diagnosis of MOC to CRS and HIPEC for PMP was 28 months (range, 0 to 120 months), but was not an independent predictor of OS after CRS and HIPEC. Despite this, we recommend a definitive follow-up schedule after CRS and HIPEC to detect recurrence and plan subsequent management. We performed patient follow-up every 6 months. Median follow-up was 31 months (range, 6 to 108 months). At the last follow-up in June 2020, 9 (41%) patients were still alive, and the median OS was 12 months (range, 1 to 102 months). 2-, 3-, and 5-year survival rates were 23, 9, and 5%, respectively, which is significantly lower than appendix-derived PMP, where 5- and 10-year OS range from 72 to 96% and 55 to 82%, respectively (15).

In our study, 10 (45%) patients with MOC-derived PMP received PSC before CRS and HIPEC, and we scored the prior surgical outcomes with PSS for each patient; PSS-0 was the rating for no surgery or biopsy, PSS-1 for surgery in one abdominal region only, PSS-2 for surgery in two to five regions, and PSS-3 for surgery in more than five regions. however, PSC and PSS were not independent predictors of OS after CRS and HIPEC. In contrast, PSC and PSS may be used as prognostic indicators in appendix derived PMP (6). Chemotherapy is indispensable for treating most EOC, but the effectiveness of adjuvant chemotherapy for MOC is not clear, as MOC are resistant to conventional platinum-based chemotherapy and respond poorly to treatment.

Serum tumor markers, including CA125, CA19-9, and CEA, are indicative of the cancerous disease process. Studies show that prechemotherapy serum levels of CA125 are significantly increased in serous ovarian cancer, but not MOC (16). The “2020 National Comprehensive Cancer Network (NCCN) Ovarian Cancer Clinical Practice Guidelines (1st Edition)” (17) recommend that CA19-9 should be used as a screening marker for MOC, and evidence suggests that serum CA19-9 may represent a novel prognosticator with potential to improve prediction of OS in patients with PMP treated with CRS and HIPEC (18). Elevated preoperative serum CEA has been strongly correlated with advanced stage in primary MOC, which may indicate a poorer prognosis (19). Despite this, in the present study, elevated serum CA125, CA19-9, and CEA were not independent predictors of OS after CRS and HIPEC in patients with MOC-derived PMP.

The combination of CRS and HIPEC is recognized as the gold standard treatment for appendix derived PMP (18, 20). As there are no alternatives specifically recommended for MOC-derived PMP, we adopted CRS and HIPEC to treat the patients included in this study. According to the univariate analysis, histopathologic subtype of MOC-derived PMP (HGMC *vs.* LGMC) was prognostic for OS after CRS and HIPEC. Among patients stratified by the histopathologic subtype of MOC-derived PMP (HGMC or LGMC), PCI (<20 *vs.* ≥20) and CCR (0/1 *vs.* 2/3) were prognostic for OS in patients with HGMC. Multivariate analysis was performed to eliminate the influence of confounding factors. Findings showed that HGMC and a high PCI (>20) were independent predictors of poor OS after CRS and HIPEC in patients with MOC-derived PMP. Consistent with this, one previous report showed that patients with HGMC-PMP registered in a PMP database had poorer 5-year OS than patients with LGMC (21). Another study showed that higher PCI predicted poorer progression-free survival in patients with appendix-derived PMP, and the influence of the PCI remained a significant prognostic variable for both patients with low-grade appendiceal pseudomyxoma and appendiceal adenocarcinoma (6). Taken together, these data suggest that early treatment with CRS and HIPEC, before tumor burden becomes high, may provide a survival benefit for patients with MOC-derived PMP.

In a previous study in appendix-derived PMP, a higher CCR score (CCR2 or CCR3) was associated with a significantly poorer 5-year survival rate of 24% compared to 85% in patients with CCR0 or 80% in patients with CCR1. The difference remained significant when stratified by histopathologic subtype (6). Consistent with this, previous findings from our hospital revealed that CCR score is a predictor of prognosis in appendix derived PMP, whereby OS was worse in patients with CCR3 compared to CCR 0/1/2 (22). In the present study, on univariate analysis, CCR (0/1 vs. 2/3) was prognostic for OS in patients with HGMC MOC-derived PMP. It is reasonable to assume that maximal cytoreduction will achieve better long-term survival, but CCR was not an independent predictor of OS on multivariate analysis in our patient population, possibly reflecting our small sample size.

Previous reports did not find any difference in survival between appendix-derived PMP and non-appendix-derived PMP (23, 24). However, the OS of non-appendix-derived PMP was lower than that of appendix-derived PMP in the present study. One of the reasons may be due to the small sample size. Eight patients with high-grade disease were dead within 1 year after operation, which greatly diluted the therapeutic effect. The other reason is that patients have a common problem of overloading small intestinal tumors. Only three patients with low-grade disease were completely cytoreduced which were still alive at the time of follow-up. The radical cure rate was low, which affects the overall prognosis. Nevertheless, we can still realize that CCR0/1 is a good prognostic factor, and for these patients with CCR0/1, early aggressive surgery should be recommended.

There are several limitations in the present study. First, the low sample size of the present study limited the power of the results. Second, more than half (12/22, 54.5%) of the included patients were transferred from local hospital with poor general condition. Therefore, the final survival was relatively low. Finally, as the MOC-derived PMP is rare, the sample size was small. Large-scale studies are needed in the future to confirm our results.

In conclusion, histopathologic subtype and PCI may be applied as predictors of prognosis in patients with MOC-derived PMP. Findings from this study will inform the management of patients with MOC-derived PMP; however, similar to most research on PMP, our study was limited by sample size. Multicenter clinical studies with large sample sizes and investigations into the molecular biology of MOC-derived PMP are required to further our understanding of this disease entity.

## Data Availability Statement

The raw data supporting the conclusions of this article will be made available by the authors, without undue reservation.

## Ethics Statement

The study was approved by the Ethic committee of Aerospace Center Hospital, Beijing, China (no.20161109-ST-07). The patients/participants provided their written informed consent to participate in this study.

## Author Contributions

The author contributions were as follows: FF and RM conceived and designed the experiments. HX and RM provided study material or patients. XM and YL collected and assembling data FF and XM analyzed and interpreted the data. FF and XM contributed to the draft of the manuscript. FF, XM, YL, RM, and HX revised the manuscript critically for important intellectual content. All authors agreed to be accountable for all aspects of the work in ensuring that questions related to the accuracy or integrity of any part of the work are appropriately investigated and resolved. All authors contributed to the article and approved the submitted version.

## Conflict of Interest

The authors declare that the research was conducted in the absence of any commercial or financial relationships that could be construed as a potential conflict of interest.
